# Synergising universal health coverage and global health security in the Western Pacific Region

**DOI:** 10.7189/jogh.15.04037

**Published:** 2025-02-14

**Authors:** Yuhua Lai, Di Liang, Albino Bobogare, Buyanjargal Yadamsuren, Esabelle Yam, Siyan Yi, Hugo Bugoro, Jiayan Huang

**Affiliations:** 1 School of Public Health, Global Health Institute, Fudan University, Shanghai, China; 2 Ministry of Health and Medical Services, Honiara, Solomon Islands; 3 Mongolian Association of Family Medicine Specialists, Ulaanbaatar, Mongolia; 4 College of Health and Medicine, Australian National University, Canberra, Australia; 5 Saw Swee Hock School of Public Health, National University of Singapore, Singapore; 6 KHANA Centre for Population Health Research, Phnom Penh, Cambodia; 7 Centre for Global Health Research, Public Health Program, Touro University California, Vallejo, California, USA; 8 Solomon Islands National University, Solomon Islands

## Abstract

**Background:**

Universal health coverage (UHC) and global health security (GHS) should be pursued synergistically to strengthen health systems. However, existing studies found that the efforts toward the two agendas were divergent worldwide. We reviewed the synergy status between UHC and GHS in the Western Pacific Region (WPR) to provide evidence for decision-makers to promote synergy.

**Methods:**

We collected the UHC service coverage index (UHC SCI) and the GHS index (GHSI) scores. We created a four-quadrant diagram to discover the gap in UHC and GHS capacities within WPR and divide WPR countries into four groups based on the global mean scores. Further, we adopted global spatial autocorrelation analysis to discover spatial aggregations of high and low scores by calculating Moran’s I. In addition, we conducted a correlation analysis to assess the synergy level in WPR and reveal the gap between Pacific Island countries or territories (PICTs) and non-PICTs. We conducted key informant interviews to uncover actual scenarios and address gaps in the quantitative evidence.

**Results:**

Compared to the global mean UHC SCI and GHSI scores, nine out of 13 non-PICTs had higher scores, while all 14 of the PICTs had lower scores for both indexes. The Moran’s I for WPR countries’ UHC SCI and GHSI scores in 2021 were 0.20 and 0.23, respectively (Z-score >2.58; *P* < 0.01). The correlation coefficients between the two index scores were 0.722 (*P* < 0.001) at the global level and 0.869 (*P* < 0.001) at WPR. Within the WPR, the correlation coefficients were 0.859 (*P* < 0.001) in the non-PICTs and –0.026 (*P* > 0.05) in the PICTs.

**Conclusions:**

The synergy level between UHC and GHS was high in the WPR, but this mainly came from the synergy in the non-PICTs. The two agendas have barely synergised the PICTs. To build a safer and healthier WPR, it is important to pay more attention to the countries that have weaker health capacities in the region and narrow the gap.

Universal health coverage (UHC) and global health security (GHS) are two critical global health agendas that share a common goal of improving population health [1,2]. UHC means providing access to comprehensive, appropriate, timely, and quality health services without financial hardship [3]. Meanwhile, GHS focuses on the proactive and reactive activities required to minimise the impact of public health emergencies that endanger people’s health across international borders [4].

The synergy between UHC and GHS should be maximised to strengthen health systems’ ability to save more lives, especially in low- and middle-income countries [4]. It helps build resilient health systems capable of sustaining the delivery of quality and basic health services, especially for the vulnerable, despite the shocks of health threats [5,6]. Furthermore, integrating efforts towards UHC and GHS helps optimise the use of resources and reduce health expenditure [7]. Generally, the synergy between UHC and GHS refers to the effects generated when an intervention, institutional capacity, or policy positively and simultaneously contributes to the continuity, quality, or expansion of routine health services and the strengthening of emergency response capabilities [1,8,9]. For example, promoting cross-sectoral collaboration is important for synergy between UHC and GHS [4,10]. Through involving various public and private sectors in decision-making processes during the COVID-19 pandemic, some countries were able to pool together resources to conduct effective disease-containing measures while ensuring all levels of essential health services were maintained [11]. More importantly, dis-synergy in existing policies and programs should be addressed to avoid duplication, fragmentation, and conflicting efforts that negatively affect either or both agendas. However, dis-synergy in pursuing UHC and GHS exists worldwide and in countries of all income levels [12–18]. The outbreak of COVID-19 has further exacerbated this long-standing issue [3,19–21].

In 2023, the Lancet Commission published a global case study on synergies between UHC, health security, and health promotion [4]. Within its overarching context, the Commission conducted case studies as its subunits through key informant interviews, desk review of literature, and stakeholder meetings [1,16,17,22,23]. Other studies have investigated similar topics using key informant interviews, literature reviews, and policy analysis [2,3,12,15,24]. Few quantitative methods were used. For example, Assefa et al. evaluated the relationship between UHC and GHS by correlation analysis to promote synergistic actions [2]. In terms of research scope, the Lancet Commission’s case studies were conducted at the national level, including in Bangladesh, Ethiopia, and nine other countries, to provide an in-depth investigation of different contextual backgrounds. Other publications focus more on general global trends or broad situations occurring worldwide. Based on the existing studies, there are two main research gaps. First, existing studies have shown limited research methods to evaluate the synergies, most of which have been conducted qualitatively. Second, all existing studies focused on specific countries or the general situation worldwide, while few studies focused on a region and considered the influences from neighbouring countries.

As one of the six World Health Organization (WHO) regions, the Western Pacific Region (WPR) includes 37 countries or areas that vary widely demographically and economically (Table S1 in the **Online Supplementary Document**). Among the 37 countries, 22 are located in the middle of the Pacific Ocean, have some of the world’s smallest land areas and populations, and are categorised as Pacific Island countries or territories (PICTs). All PICTs share some key characteristics, including geographic isolation, high incidence of communicable diseases and non-communicable diseases (NCD), and vulnerability to climate change and natural disasters. In addition, the PICTs either have relatively shorter independence years after colonisation or are currently territories of developed countries, which leads to high reliance on external assistance for development. These characteristics leave the PICTs constrained health resources and infrastructure and weaker health capacities compared to other non-PICTs in the region, which could affect their capacities to pursue UHC and GHS synergistically. To provide evidence for global and country-level decision-makers in fostering resilient health systems, in this study, we aimed to quantitatively explore the status of synergy between UHC and GHS in the WPR.

## METHODS

### Data source

Developed by the WHO and World Bank, the UHC service coverage index (UHC SCI) is an index that measures the coverage of essential services [25]. It is a geometric mean calculated from four sub-indexes – reproductive, maternal, newborn, and child health (RMNCH), infectious diseases (ID), NCD, and service capacity and access (SCA). The RMNCH sub-index measures the service utilisation for family planning, antenatal care, diphtheria-tetanus-pertussis immunisation, and care-seeking for suspected pneumonia. The ID sub-index reflects the service coverage of tuberculosis treatment, HIV therapy, insecticide-treated nets for malaria prevention, and water, sanitation and hygiene. The NCD sub-index reflects the prevalence of treatment for hypertension, the mean fasting plasma glucose, and smoking prevalence. The SCA measures hospital bed density, health worker density, and the attainment of International Health Regulations core capacities [25]. All indicators scale from zero to 100. As of May 2024, UHC SCI data were issued in 2000, 2005, 2010, 2015, 2017, 2019, and 2021, so we collected all existing data [26].

The GHS index (GHSI) is a comprehensive index developed through a partnership between the Nuclear Threat Initiative, the Johns Hopkins University Centre for Health Security, and The Economist Intelligence Unit. It assesses a country’s capability to prevent, detect, and respond to public health emergencies. Its score is a weighted sum of six category scores. The prevention category assesses countries’ actions to prevent the emergence or release of pathogens through six indicators. The detection category reflects the early detection and reporting of epidemics of potential international concern through six indicators. The response category measures rapid response to and mitigation of the spread of an epidemic through seven indicators. The health system category evaluates the attainment of a sufficient and robust health system to treat the sick and protect health workers through seven indicators. The compliance category reflects countries’ commitments to improving national capacity, financing plans to address gaps, and adherence to global norms through six indicators. The risk category reflects countries’ risk environment and vulnerability to biological threats through five indicators [27]. The scores also ranged from zero to 100. GHSI data were first issued in 2019 and updated in 2021, so we collected data from both years [28].

There are 37 countries or territories in the WPR. We included only 27 in this study due to the limited data availability from the following ten territories: American Samoa, French Polynesia, Guam, Macao, Hong Kong, New Caledonia, Wallis and Futuna, Northern Mariana Islands, Pitcairn Island, and Tokelau. Since they only account for a relatively small part of the region, we believe that the lack of data on them would not affect the results of this study significantly. (Table S1 in the **Online Supplementary Document**).

Among the 27 WPR countries or territories, WHO categorised the following 14 as PICTs: Cook Islands, Fiji, Federated States of Micronesia, Kiribati, Marshall Islands, Nauru, Niue, Palau, Papua New Guinea, Samoa, Solomon Islands, Tonga, Tuvalu, and Vanuatu. The other 13 countries or areas were referred to as non-PICTs in this study.

### Data analysis

#### Descriptive analysis

We organised the UHC SCI and GHSI scores in two ways. First, we graphed scatter plots to illustrate the overall performance and trend of scores and the comparison between PICTs and non-PICTs. Second, we drew a four-quadrant diagram, with the global mean UHC SCI and GHSI scores being the x- and y-axis, to divide the 27 WPR countries into four groups: 1) both UHC and GHS capacities were stronger than the global means, 2) only UHC capacity was stronger, 3)only GHS capacity was stronger, and 4) both UHC and GHS capacities were weaker than the global means.

#### Global spatial autocorrelation analysis

Adjacent countries are likely to impact each other’s achievement in UHC and GHS. Therefore, to determine whether countries with stronger and weaker health capacities tend to cluster in specific areas in the WPR, we calculated the global spatial autocorrelation index (Moran’s I) through ArcMap, version 10.2 (Environmental Systems Research Institute, Redlands, California, USA) [29]. More importantly, it helps identify which countries require more attention to ensure regional safety.

Moran’s I is an index commonly used to explore spatial dependence on data. While several software tools, such as R, Python, and MATLAB, can be used to calculate Moran’s I, we chose ArcMap due to its widespread use in academic publications and user-friendly interface. The values for Moran’s I range from –1 to 1, where ‘–1’ indicates ‘perfectly dispersed,’ and ‘1’ means ‘perfectly clustered’ [30,31]. We calculated a Z-score and *P*-value along with Moran’s I to evaluate the significance of the index. Z-scores are standard deviations. A Z-score and a *P*-value tell whether the null hypothesis can be rejected. When rejected, it indicates that the values exhibit statistically significant clustering or dispersion rather than a random pattern. When Moran’s I is >0, with Z-score >2.58 and *P* < 0.01, it indicates a 99% chance that positive spatial aggregation exists in this region.

#### Correlation analysis

Synergy occurs when an effort works towards achieving UHC and GHS simultaneously. Similarly, when UHC progresses as GHSI improves, or the other way around, synergy is likely. Therefore, if there is synergy between the two agendas, their correlation should be fairly strong. We calculated Pearson correlation coefficients (r) to measure the correlation between UHC and GHS in the WPR.

To assess the correlations between UHC SCI and GHSI, UHC SCI and GHSI sub-indexes, GHSI and UHC SCI sub-indexes, and GHSI sub-indexes and UHC SCI sub-indexes for WPR countries in 2021 and calculate correlation coefficients, we used SPSS, version 27 (International Business Machines Corporation, New York, New York, USA). We set a confidence level of 95%.

To compare the synergy level between UHC and GHS in WPR with the other five WHO regions and the globe, we calculated the correlation coefficients between UHC SCI and GHSI for Eastern Mediterranean, Americas, Europe, Africa, South-East Asia regions, and the world. In addition, to determine the gap between PICTs and non-PICTs, we calculated correlation coefficients between UHC SCI and GHSI in PICTs and non-PICTs.

#### Key informant interviews

We conducted key informant interviews to uncover actual scenarios in the WPR countries and address gaps in the evidence from the quantitative analysis. We employed a purposive sampling strategy to select informants with extensive experience or knowledge of the topics examined. The respondents included decision-makers who are current or former senior officials from Ministries of Health and other national agencies, researchers from national universities, and officials from international organisations who have worked in WPR countries. To ensure diverse representation, the selection criteria considered the variety of contexts within the WPR. This included knowledge of countries with differing population sizes (using one million as the median dividing point), geographic locations encompassing PICTs and non-PICTs, and medium to lower UHC SCI and GHSI scores.

We designed the interview topic guide after a thorough review of existing literature on the synergy between global health agendas and insights derived from the quantitative analysis. Ultimately, ten key informants from Australia, Cambodia, China, Mongolia, the Solomon Islands, and Vanuatu were included in this study. We obtained informed consent from all interviewees included in this study.

## RESULTS

### Overview of the UHC SCI and GHSI scores in the WPR

**Figure 1:**
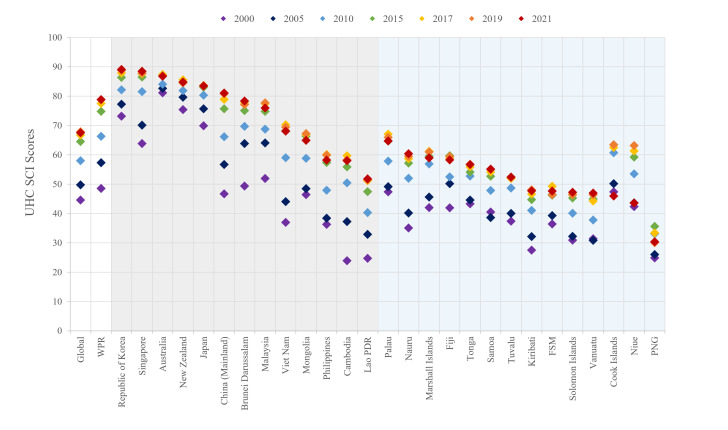
The UHC SCI scores of WPR countries or areas. FSM – Federated States of Micronesia, Lao PDR – Lao People’s Democratic Republic, PICTs – Pacific Island countries or territories, PNG – Papua New Guinea, UHC SCI – universal health coverage service coverage index, WPR – Western Pacific Region.

Overall, the UHC SCI scores in the WPR have increased since the index was established in 2000 (**Figure 1**). Compared to the other WHO regions, the WPR was ranked third in 2021 (UHC SCI = 78.79), following the European Region (UHC SCI = 81.06) and the Region of the Americas (UHC SCI = 80.02). The WPR also scored higher than the global means in all the years in which UHC SCI scores were calculated. However, although the whole region appeared to perform relatively well, the regional gap had been substantial. In 2021, the difference between the highest score (UHC SCI = 89.09 in the Republic of Korea) and the lowest (UHC SCI = 30.39 in Papua New Guinea) was 58.7. More importantly, there was an apparent gap between non-PICTs’ and PICTs’ performances. Ten of 13 non-PICTs scored >60 in 2021, while 11 out of 14 PICTs scored below.

**Figure 2:**
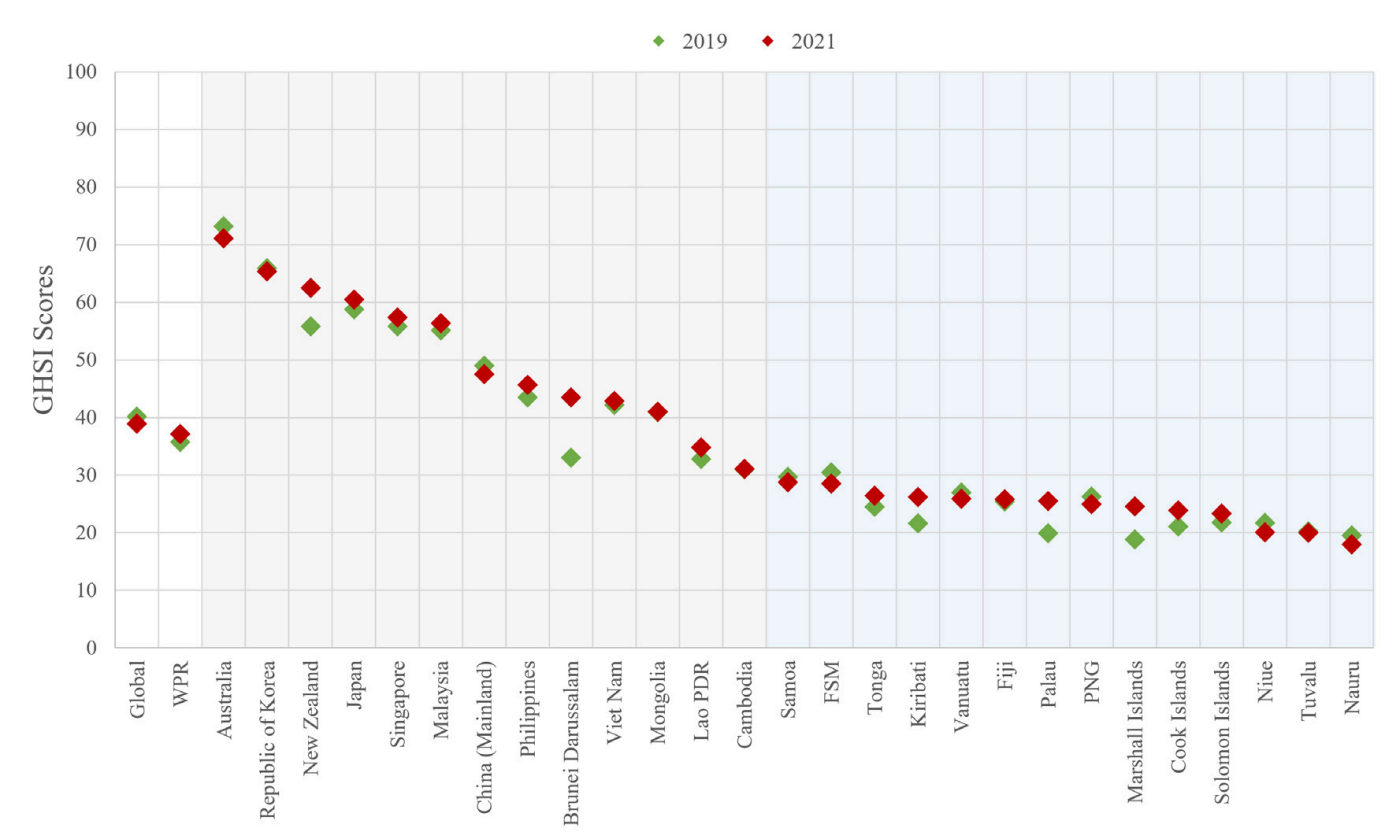
The GHSI scores of WPR countries or areas. GHSI – global health security index, FSM – Federated States of Micronesia, Lao PDR – Lao People’s Democratic Republic, PICTs – Pacific Island countries or territories, PNG – Papua New Guinea, WPR – Western Pacific Region.

Regarding the GHSI scores, WPR’s mean scores were lower than the global mean in 2019 and 2021 (**Figure 2**). In addition, the differences between the highest and the lowest GHSI scores were tremendous, reaching over 50 points in both years. From the distribution of GHSI scores, the huge gap between non-PICTs and PICTs is shown explicitly. In 2021, non-PICTs’ GHSI scores ranged from nearly 30 to >70 points. However, all 14 PICTs scored <30 in the same year. These indicated significant GHS gaps within WPR, leaving the region vulnerable to global health emergencies.

### Four groups of the UHC and GHS capacities

**Figure 3:**
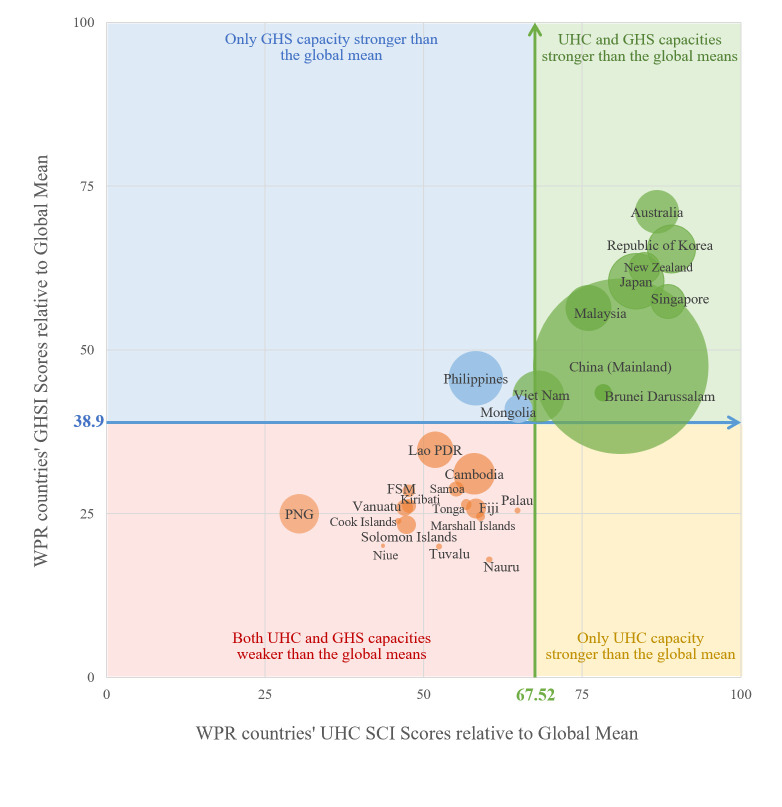
The four-quadrant diagram of WPR countries or areas with different combinations of the UHC and GHS capacities in 2021. The global mean UHC SCI score in 2021 was 67.52. The global mean GHSI score in 2021 was 38.9. Countries in the green quadrant had UHC and GHS capacities stronger than the global mean. Countries in the yellow quadrant had UHC capacity stronger but GHS capacity weaker than the global mean. Countries in the blue quadrant had GHS capacity stronger but UHC capacity weaker than the global mean. Countries in the red quadrant had both UHC and GHS capacities weaker than the global mean. The size of the circles represents the population size of the country or territory. GHS – global health security, FSM – Federated States of Micronesia, Lao PDR – Lao People’s Democratic Republic, PNG – Papua New Guinea, UHC – universal health coverage, WPR – Western Pacific Region.

In the four-quadrant diagram, which shows four groups of UHC and GHS capacities in comparison to the global means, almost all 27 WPR countries are in the first and third quadrants, except for the Philippines and Mongolia, which are in the second quadrant (**Figure 3**). No WPR country is in the fourth quadrant.

The first quadrant indicates stronger UHC and GHS capacities compared to the global mean. Nine countries are in this quadrant, including Australia, the Republic of Korea, and New Zealand, and all are non-PICTs. Except for China (Mainland), Malaysia, and Vietnam, the other six are all high-income countries.

The second quadrant includes the countries with stronger GHS but weaker UHC capacity than the global mean, the Philippines and Mongolia. The two countries are both lower-middle-income countries. Although located in the second quadrant, their UHC SCI scores are close to the global mean (UHC SCI = 58.21 in the Philippines and UHC SCI = 64.95 in Mongolia), making them fairly close to the first quadrant.

The third quadrant indicates weaker UHC and GHS capacities relative to the global mean. Nearly 60% of WPR countries (n = 16/27) are located in this quadrant, showing that the region has weaker health capacities. Of the 16 countries, 14 are PICTs, except for the Lao People’s Democratic Republic and Cambodia.

### Spatial correlation of the UHC SCI and GHSI

The Moran’s I for UHC SCI and GHSI scores in 2021 were 0.20 and 0.23, respectively (Z-score >2.58; *P* < 0.01). This indicates a 99% chance positive spatial aggregations existed in the WPR, meaning hot and cold spots could exist. A hot spot indicates that a country had a relatively high score and was close to other high-score countries. A cold spot implies the opposite.

Based on the geographic distribution of the high UHC SCI scores, it is likely that some hot spots of UHC SCI would appear in the Republic of Korea, Japan, China (Mainland), and Mongolia, and some would gather in Malaysia, Singapore, Brunei Darussalam, and Vietnam (Figure S1 in the **Online Supplementary Document**). On the other hand, some cold spots would likely appear in the Western part of the Pacific area, near Papua New Guinea. In terms of the spatial distribution of GHSI scores, it appeared that no hot spot might exist since there were few high scores, and they were not closely located (Figure S2 in the **Online Supplementary Document**). However, several cold spots would likely gather in the Pacific Islands, especially near Nauru and Tuvalu.

### Correlation between the UHC SCI, GHSI, and their sub-indexes

The correlation coefficient between UHC SCI and GHSI in the WPR was r = 0.869 (*P* < 0.001), which was much higher than the globe (r = 0.722; *P* < 0.001) and the other five regions: Eastern Mediterranean (r = 0.703; *P* < 0.001), Americas (r = 0.577; *P* < 0.001), Europe (r = 0.506; *P* < 0.001), Africa (r = 0.400; *P* < 0.001), and South-East Asia (r = 0.423; *P* > 0.05).

Within the WPR, the correlation coefficients between UHC SCI and GHSI sub-indexes were r = 0.85 (*P* < 0.001) for prevention, r = 0.83 (*P* < 0.001) for detection, r = 0.82 (*P* < 0.001) for health systems, r = 0.79 (*P* < 0.001) for risk, r = 0.72 (*P* = 0.001) for response, and r = 0.70 (*P* = 0.001) for compliance. On the other hand, the correlation coefficients between GHSI and UHC SCI sub-indexes were r = 0.84 (*P* < 0.001) for NCD, r = 0.71 (*P* < 0.001) for RMNCH, r = 0.69 (*P* < 0.001) for SCA, and r = 0.59 (*P* = 0.001) for ID. All the correlations indicated strong relationships. In addition, all sub-indexes also showed significant correlations with each other (Table S2 in the **Online Supplementary Document**).

Comparing the non-PICTs and PICTs, the correlation coefficient between UHC SCI and GHSI was r = 0.859 (*P* < 0.001) in the non-PICTs and r = –0.026 (*P* > 0.05) in the PICTs. This showed that the high correlation in the WPR was mainly contributed by the non-PICTs. The relationship between the two agendas in the PICTs appeared to be insignificant.

Looking at the correlation between the UHC SCI and GHSI sub-indexes in non-PICTs, the correlation coefficients were r = 0.838 (*P* < 0.001) for risk, r = 0.824 (*P* = 0.001) for response, r = 0.821(*P* = 0.001) for report and prevention, r = 0.816 (*P* = 0.001) for health systems, and r = 0.439 (*P* = 0.133) for compliance. All GHSI sub-indexes had strong relationships with UHC SCI, except for the compliance sub-index. In addition, GHSI was also highly correlated with all UHC SCI sub-indexes. The correlation coefficients were r = 0.789 (*P* = 0.001) for SCA, r = 0.694 (*P* = 0.008) for NCD, r = 0.686 (*P* = 0.01) for RMNCH, and r = 0.648 (*P* = 0.017) for ID.

Contrary to non-PICTs, the relationships between the main indexes and sub-indexes in PICTs were mostly insignificant. The only significant correlation was between GHSI and the NCD sub-index of UHC SCI. However, it was a negative correlation (r = –0.646; *P* = 0.013).

## DISCUSSION

Based on our findings, the relationship between UHC and GHS in the WPR was much stronger than the globe and all other five regions, indicating a high level of synergy in the region. However, this mainly came from the synergy in the non-PICTs. The two agendas were in fact barely synergized in the PICTs.

To explore the potential key synergy factors between UHC and GHS in WPR, we compared the policies, actions, and actors in the PICTs and non-PICTs. Among all the factors identified, the country’s implementation capacity was agreed upon by all involved key informants as one of the key determinants. This capacity could be further determined by health financing and health workforce capacity.

### Health financing sources can affect intersectoral collaboration and, therefore, the synergy

Health funds are mainly sourced from public taxation and compulsory health insurance schemes in the countries that successfully synergise UHC and GHS, which are mostly high-income countries. For example, New Zealand funds its health system through general taxation, which supports a wide array of public health services and ensures accessibility for all citizens [32]. The Republic of Korea operates the National Health Insurance Service, a mandatory health insurance scheme that requires contributions from all employed citizens and employers, supplemented by government support, to ensure comprehensive health coverage [33]. With full control of the funds, the governments can allocate the funds based on the countries’ health priorities related to both preventive health care and emergency preparedness. During the COVID-19 pandemic, New Zealand adopted an all-of-government strategy to encourage different sectors to work together, ensuring strict disease containment and continuous delivery of essential health services [34]. Similarly, the Republic of Korea demonstrated the effectiveness of a whole-of-government approach supported by its UHC system [35]. Through this approach, the country was able to integrate GHS measures into UHC to ensure that COVID-19 testing and treatment were accessible without financial barriers [36].

However, countries with difficulty synergising the two agendas rely heavily on development partners to support their health actions financially. The development partners usually fund specific disease controls or specific programs, which could lead to the fragmentation of the health system. For example, in Papua New Guinea, the Global Fund funds malaria and tuberculosis control programs, while the Australian Department of Foreign Affairs and Trade provides significant resources for maternal and child health initiatives. In order to obtain financial assistance from the development partners, the countries need to demonstrate effective use of funds and progress towards predefined health targets set by these partners. Therefore, different sectors or departments would focus on solving specific health issues instead of considering those interrelated issues as a whole. This would make it challenging for them to collaborate with each other to pursue synergy. To promote intersectoral collaboration, it could be beneficial for PICTs to develop a sector-wide approach to align external funding priorities with integrated health system goals. One Health or whole-of-government approach within countries could also improve resource-sharing mechanisms and promote synergy.

### A sufficient and competent health workforce can be the basis for synergising UHC and GHS

To promote multisectoral cooperation and implement UHC and GHS actions and policies as designed by the government, sufficient, sophisticated, and well-distributed health workforce are crucial. Take the countries with stronger UHC and GHS capacities as examples. Australia has invested heavily in its health care workforce through comprehensive education and training programs, as well as through initiatives that encourage health care professionals to work in rural and remote areas [37]. Japan also focuses on advanced health care workforce strategies and interdisciplinary education programs that prepare health care workers to address both routine health care needs and emergency responses efficiently [38].

However, in the PICTs, geographical isolation, limited local training facilities, and the high emigration rate of trained professionals have resulted in the lack and uneven distribution of trained health workers, making it difficult to enforce the policies as designed [39,40]. Limited local training facilities force reliance on medical education institutions abroad, such as in Fiji, Papua New Guinea, and Australia, which cannot produce enough clinicians to meet the region’s needs [40]. This challenge is exacerbated by the geographical realities of the PICTs. The large number of dispersed inhabited islands and predominantly rural populations make transportation of medical supplies costly and inconvenient. Insufficient incentives for health workers, such as inadequate accommodation for them and their families in remote areas, further contribute to workforce shortages in underserved regions [41]. Without sufficient personnel in the areas where they are most needed, it becomes difficult for populations to access the care and protection they require, which hinders progress in achieving both UHC and GHS. To address these workforce challenges, it is crucial for the PICTs to establish policies or plans that help foster and retain health care professionals through building local training facilities, offering career advancement opportunities, or designing incentive schemes.

### Stronger GHS alone is insufficient to protect people from health emergencies

Mongolia and the Philippines exhibit a unique profile in the WPR, demonstrating stronger GHS capacities but weaker UHC achievements compared to global means. Early in the pandemic, both countries implemented decisive measures to strengthen their GHS response. Mongolia activated the State Emergency Commission, imposed strict border controls, and halted international travel, supported by widespread testing and community-wide public health initiatives [42]. Similarly, the Philippines established robust containment strategies, including lockdowns, localised quarantine facilities, and coordinated actions by the Inter-Agency Task Force on Emerging Infectious Diseases [43].

Despite these efforts, both countries recorded high COVID-19 cases and deaths, with Mongolia reporting over one million cases and the Philippines exceeding four million [44]. Limited health service coverage significantly contributed to this outcome. In Mongolia, rural populations face challenges accessing health care, with shortages of essential medicines and diagnostic tools hindering timely care [45]. In the Philippines, health care inequities, overwhelmed facilities, and reliance on out-of-pocket payments left many without adequate care [46,47]. These underscore the critical need to integrate GHS and UHC systems, as strong GHS measures alone cannot compensate for gaps in health care access during crises.

### The possibility for the use of correlation analysis in this study

In selecting correlation analysis, we intend to provide an initial insight into the degree to which UHC and GHS progress in tandem across countries. We believe this foundational knowledge is valuable as it offers a crucial first step in assessing and promoting the synergy between UHC and GHS. Additionally, the choice of correlation analysis is supported by established methodologies in similar research contexts. For instance, Assefa et al. used correlation analysis to evaluate the relationship between UHC and GHS at a global level, aiming to promote synergistic actions [2]. This study aligns with their research approach while focusing specifically on the WPR. As the previous studies used earlier data before the COVID-19 pandemic, our study provides an opportunity to update the findings with more recent data, allowing for an assessment of synergy during the pandemic.

### Limitations

In this study, we used UHC SCI and GHSI as main indexes to discover the synergy status between UHC and GHS in the WPR. Although international authoritative institutions developed the two indexes, the index scores were formed based on publicly available data and estimates reported by countries. Therefore, differences in data collection, availability, and calculation methods could alter data quality [48,49]. For instance, while non-PICTs tend to have more comprehensive health information systems to reflect the real situation in the countries, the weaker data reporting and analysing capacities of PICTs might lead to delays or inaccuracy in data. Due to these differences, potential bias might exist between the situation reflected by the index scores and the actual performances of the health systems. Another limitation might exist due to the lack of data on ten territories. However, we believe that they would not affect the results of this study significantly due to the relatively small population sizes.

Lastly, there is a potential risk that correlation analysis may not fully capture the multifaceted nature of health systems or the numerous external factors influencing each country’s performance. While there are currently no other reliable methods to quantitatively measure the relationship between UHC and GHS, correlation analysis could still be a valuable exploratory tool despite its potential biases.

## CONCLUSIONS

The synergy level between UHC and GHS in the WPR was relatively high compared to other regions. However, this mainly reflected the high synergy level in non-PICTs. In fact, the PICTs had barely synergised the two agendas. The PICTs also had the weakest UHC and GHS capacities in the region. Therefore, to build a safer and healthier WPR, it is important to pay more attention to the countries that have weaker health capacities in the region and narrow the gap.

## Additional material


Online Supplementary Document
