# Supplementary material for: Synergising universal health coverage and global health security in the Western Pacific Region
Source: J Glob Health. 2025 Feb 14;15:04037. doi: 10.7189/jogh.15.04037 (PMC11827627; doi:10.7189/jogh.15.04037)
Supplement: Online Supplementary Document [file jogh-15-04037-s001.pdf]

Supplement to: Lai Y, Liang D, Bobogare A, Yadamsuren B, Yam E, Yi S, Bugoro H, Huang J. Synergising universal health coverage and global health security in the Western Pacific Region. J Glob Health. 2024;15:04037.

Table S1: Contextual indicators of WPR countries and territories

No.	Country/Territory	Population	Income Level	GDP per Capita (US dollars)	Under-five Mortality Rate	Life Expectancy at Birth	Human Development Index
1	China (Mainland)	1,425,671,352	UMI	12662.58	6.60	78.6	0.788
2	Japan	123,294,513	HI	34017.27	2.28	84.8	0.920
3	Philippines	117,337,368	LMI	3499.11	27.50	72.2	0.710
4	Viet Nam	98,858,950	LMI	4179.01	20.35	74.6	0.726
5	Republic of Korea	51,784,059	HI	32394.68	2.77	84.0	0.929
6	Malaysia	34,308,525	UMI	11993.19	7.84	76.3	0.807
7	Australia	26,439,112	HI	65077.68	3.76	83.6	0.946
8	Cambodia	16,944,826	LMI	1759.61	23.70	69.9	0.600
9	Papua New Guinea	10,329,931	LMI	3115.92	41.37	66.0	0.568
10	Lao PDR	7,633,779	LMI	2054.43	40.38	69.0	0.620
11	Hong Kong (China)	7,442,734	HI	48828.12	/	84.3	0.956
12	Singapore	6,014,723	HI	88428.70	2.15	84.1	0.949
13	New Zealand	5,228,100	HI	48216.51	4.63	/	/
14	Mongolia	3,447,157	LMI	5045.50	13.41	72.7	0.741
15	Fiji	936,376	UMI	5356.16	28.21	68.3	0.729
16	Solomon Islands	740,425	LMI	2162.67	18.28	70.7	0.562
17	Macao (China)	713,912	HI	35192.53	/	/	/
18	Brunei Darussalam	452,524	HI	37152.48	9.74	74.6	0.823
19	Vanuatu	334,506	LMI	3128.54	18.17	70.5	0.614
20	New Caledonia (France)	289,870	HI	35745.85	/	/	/
21	French Polynesia (France)	281,118	HI	18984.85	/	/	/
22	Samoa	225,681	LMI	3745.56	16.11	72.6	0.702
23	Guam (USA)	166,506	HI	40227.28	/	/	/
24	Kiribati	133,515	LMI	/	56.44	67.7	0.628
25	FSM	115,224	LMI	3766.51	24.21	70.9	0.634
26	Tonga	107,773	UMI	4681.68	10.93	/	/
27	American Samoa (USA)	47,521	HI	19673.39	/	/	/
28	Northern Mariana Islands (USA)	45,143	HI	/	/	/	/
29	Marshall Islands	41,996	UMI	6225.18	28.64	65.1	0.731
30	Palau	18,058	UMI	13421.97	22.34	65.4	0.797
31	Cook Islands	17,044	/	/	6.94	/	/
32	Nauru	12,780	HI	12142.77	26.49	64.0	0.696
33	Tuvalu	11,396	UMI	5221.53	20.37	64.9	0.653
34	Wallis and Futuna (France)	11,370	/	/	/	/	/
35	Tokelau	2,397	/	/	/	/	/
36	Niue	1,935	/	/	24.82	/	/
37	Pitcairn Islands (UK)	50	/	/	/	/	/

Note:
The countries are ordered by the population. The Pacific Island countries or territories are marked blue. Population is the data of 2023 sourced from UNICEF database. The population of Pitcairn Islands is sourced from the Pacific Community. Hong Kong and Macao account for 0.42% of population in the WPR and in the non-PICTs. The eight Pacific Island territories excluded in this study (American Samoa, French Polynesia, Guam, New Caledonia, Wallis and Futuna, Northern Mariana Islands, Pitcairn Island, and Tokelau) represent 0.04% of population in the WPR and 6.08% in the

PICTs. Income levels were classified in 2023 by World Bank. GDP per capita is the data of 2022 sourced from the World Bank. Under-five mortality rate is the data of 2022 sourced from WHO database. Life expectancy at birth and human development index are the data of 2022 sourced from United Nations Development Program. "/" means data not available. WPR – Western Pacific Region; Lao PDR – Lao People's Democratic Republic; FSM – Federated States of Micronesia; GDP – Gross Domestic Product; HI – High-income economies with a gross national income per capita of $13,846 or more; UMI – Upper middle-income economies with a gross national income per capita between $4,466 and $13,845; LMI – Lower middle-income economies with a gross national income per capita between $1,136 and $4,465.

Figure S1: The spatial distribution of UHC SCI scores of WPR countries or areas in 2021

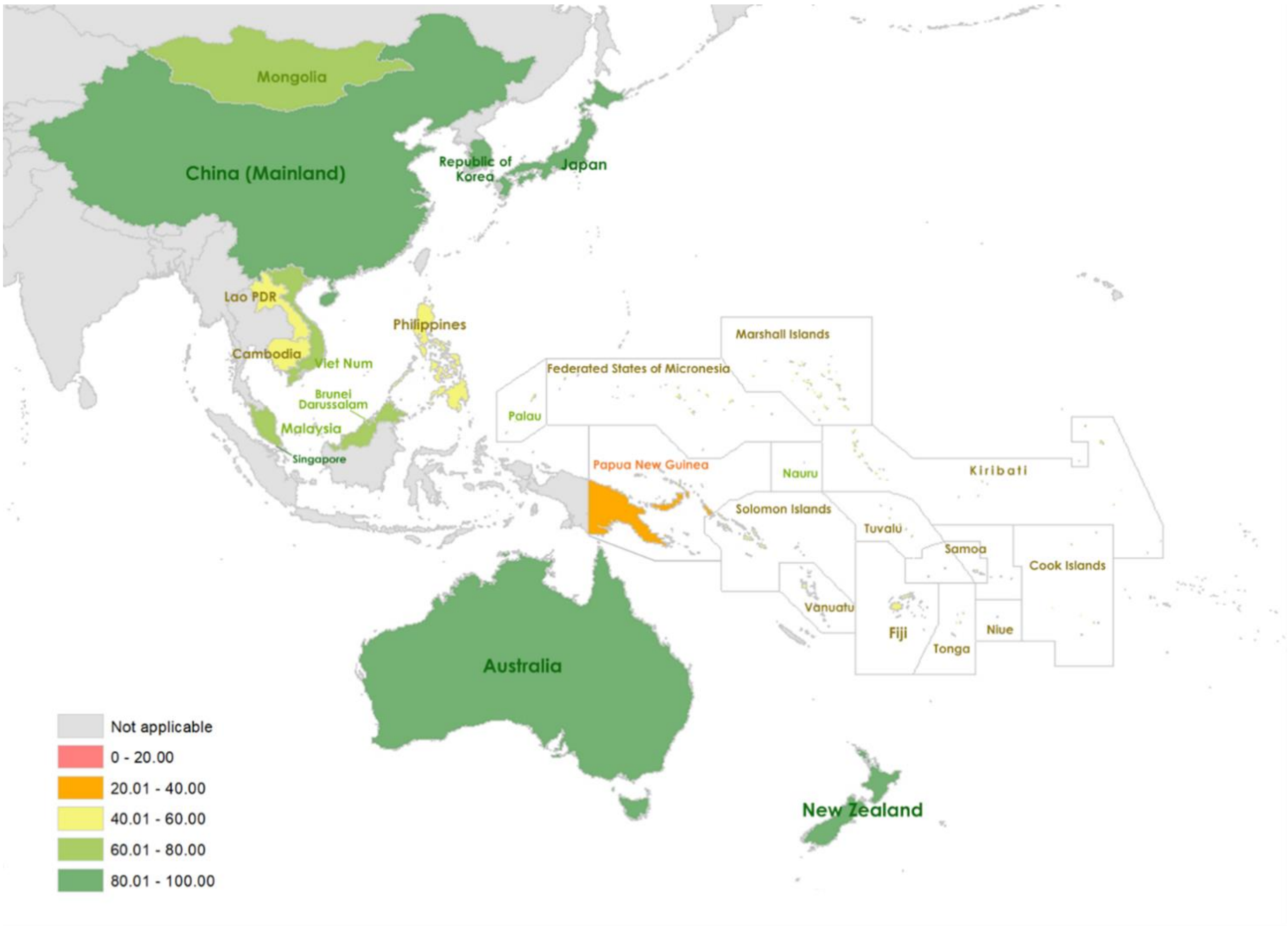

Note:

Lao PDR – Lao People's Democratic Republic; UHC SCI – universal health coverage service coverage index; WPR – Western Pacific Region.

Figure S2: The spatial distribution of GHSI Scores of WPR countries or areas in 2021

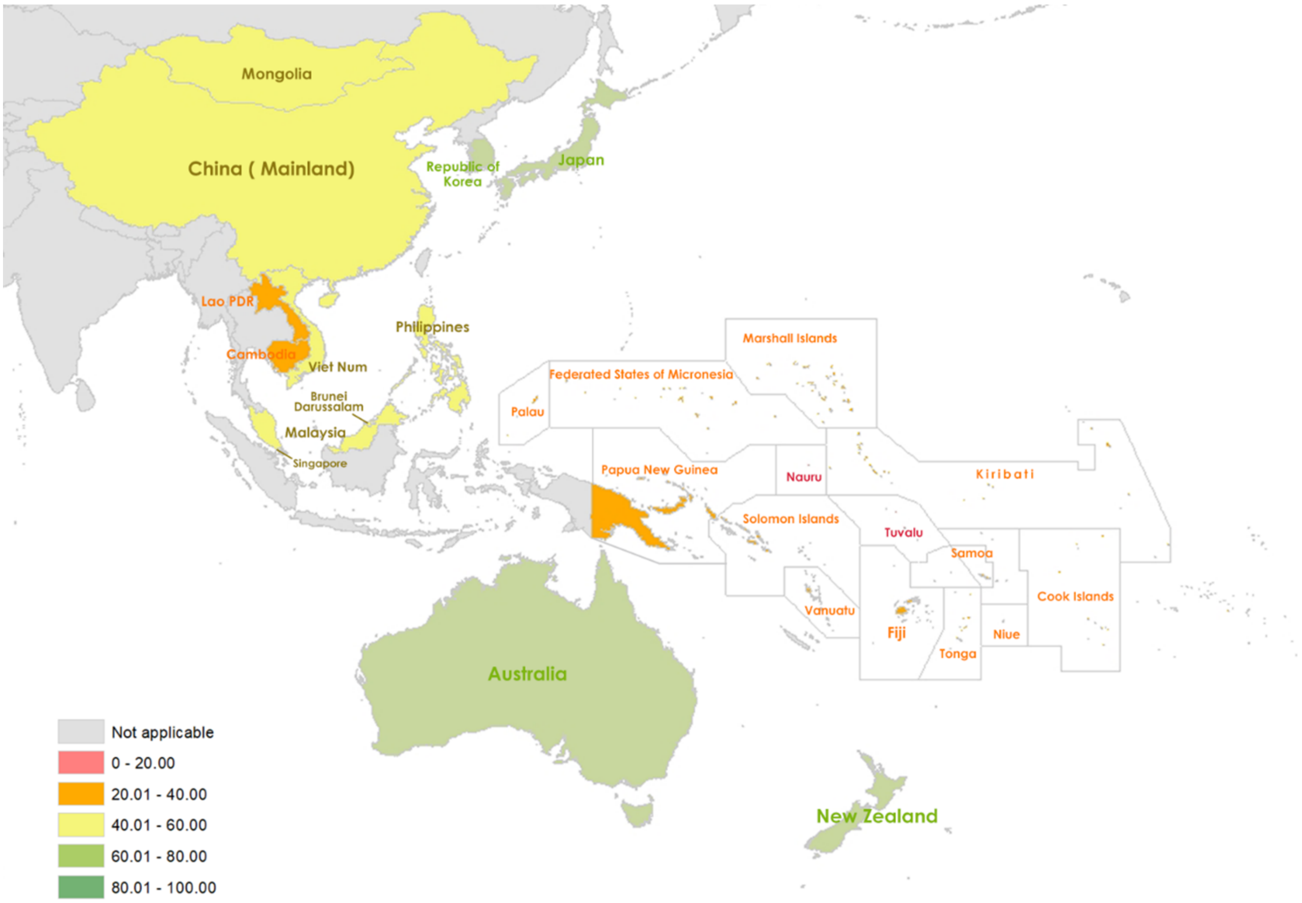

Note:
Lao PDR – Lao People's Democratic Republic; GHSI – global health security index; WPR – Western Pacific Region.

Table S2: Pearson correlation coefficients between UHC SCI sub-indexes and GHSI sub-indexes in WPR in 2021

UHC SCI Sub-indexes	GHSI sub-indexes					
	PREVENTION	DETECTION	RESPONSE	HEALTH SYSTEM	COMPLIANCE	RISK
UHC-RMNCH						
Pearson Correlation	0.732**	0.656**	0.628**	0.661**	0.502**	0.746**
Significant Level (2-tailed)	<0.001	<0.001	<0.001	<0.001	0.008	<0.001
UHC-NCD						
Pearson Correlation	0.849**	0.846**	0.604**	0.817**	0.724**	0.567**
Significant Level (2-tailed)	<0.001	<0.001	<0.001	<0.001	<0.001	0.002
UHC-ID						
Pearson Correlation	0.547**	0.572**	0.496**	0.542**	0.528**	0.489**
Significant Level (2-tailed)	0.003	0.002	0.008	0.004	0.005	0.01
UHC-SCA						
Pearson Correlation	0.667**	0.618**	0.656**	0.622**	0.457*	0.836**
Significant Level (2-tailed)	<0.001	<0.001	<0.001	<0.001	0.017	<0.001

Note:
**Correlation is significant at 0.01 level (2-tailed). *Correlation is significant at the 0.05 level (2-tailed). UHC SCI – universal health coverage service coverage index; GHSI – global health security index; WPR – Western Pacific Region; RMNCH – reproductive, maternal, newborn, and child health; NCD – non-communicable diseases; ID – infectious diseases; SCA – service capacity and access.
